# Caffeine, skeletal muscle signalling, and exercise adaptation: a narrative review separating acute ergogenic effects from chronic remodelling

**DOI:** 10.3389/fphys.2026.1875283

**Published:** 2026-06-05

**Authors:** Zhifeng Liu, Huakun Zheng, Hengzhi Deng, Mingrui Wang, Wenxin Du, Ming Chen, Zike Zhang, Bitai Wu

**Affiliations:** 1 Hengyang Normal University, Hengyang, China; 2 Shanghai University of Sport, Shanghai, China; 3 Universiti Malaya, Kuala Lumpur, Malaysia; 4 Beijing Sport University, Beijing, China; 5 Zhejiang University, Hangzhou, China; 6 Kunming University of Science and Technology, Kunming, China; 7 Shaanxi Normal University, Xi’An, China

**Keywords:** AMPK, caffeine, mitochondrial biogenesis, molecular signalling, narrative review, PGC-1α, skeletal muscle adaptation, training quality

## Abstract

Caffeine is one of the most widely used and extensively studied ergogenic aids in sport, yet whether its well-established acute benefits extend to the chronic remodelling of skeletal muscle remains unresolved. In this narrative review, we distinguish the acute ergogenic effects of caffeine from its potential influence on chronic skeletal muscle remodelling, because the mechanisms that improve acute performance need not be those that govern repeated tissue adaptation. The evidence for acute ergogenicity rests on a large human literature, in which adenosine receptor antagonism is the probable dominant mediator, with additional contributions from potassium handling, ryanodine receptor 1 (RyR1) sensitisation, altered contractile behaviour, and reduced perceived effort. Evidence that repeated caffeine exposure around exercise modifies chronic skeletal muscle adaptation in humans remains limited; available training trials are short, narrow in modality, and lack muscle biopsy endpoints, and several acute endurance studies under substrate restriction are better interpreted as training quality mediator evidence. Preclinical work is more mechanistic but directionally mixed, supporting Ca²^+^ linked CaMKKβ (Ca²^+^/calmodulin-dependent protein kinase kinase β) and AMP-activated protein kinase (AMPK) signalling, autophagy, peroxisome proliferator-activated receptor gamma coactivator 1-alpha (PGC-1α) related transcription, and mitochondrial quality control on one side, and raising an attenuation hypothesis through protein synthesis, recovery, and tissue-specific remodelling on the other. We therefore evaluate three working models in which caffeine acts as an amplifier, a partial mimic, or an attenuator of exercise-related signalling. Current human evidence is most compatible with partial mimicry and training quality mediation rather than direct amplification or impairment of long-term tissue adaptation. Resolving this question will require human training studies that combine muscle biopsy endpoints, caffeine-abstinent post-training testing, objective sleep monitoring, and explicit control of external training load. On balance, the current evidence supports interpreting caffeine as a reliable acute ergogenic aid and a plausible mediator of training quality, rather than as a proven direct modifier of chronic skeletal muscle adaptation.

## Introduction

Caffeine occupies a distinctive position in exercise physiology (Graham, 2001; Spriet, 2014). The compound has been examined across endurance, intermittent, and resistance exercise, and acute ergogenicity is consistent enough to support consensus recommendations for acute use (Graham, 2001; Grgic et al., 2020; Guest et al., 2021; Maughan et al., 2018). The mechanistic explanation has shifted over the decades. Early work emphasised fatty acid mobilisation and glycogen sparing (Costill et al., 1978; Spriet et al., 1992). Subsequent studies placed greater weight on the central nervous system, perceived effort, membrane excitability, ion handling, and Ca²^+^ linked contractile behaviour (Graham et al., 2000; Meeusen et al., 2013; Spriet, 2014). The acute performance effect is now reasonably well described. The relative contribution of central, systemic, and skeletal muscle mechanisms remains debated.

The chronic question is different. Skeletal muscle adaptation here refers to durable changes in muscle phenotype after repeated exercise exposure. The relevant outcomes include mitochondrial respiration, mitochondrial content, mitochondrial quality control, protein turnover, contractile phenotype, capillarisation, and neuromuscular function (Egan and Zierath, 2013; Hood, 2019). They differ from transient signalling responses, acute changes in force, and session level work output. A supplement can improve the performance of a single session without altering the tissue biology that governs adaptation over weeks (Hawley et al., 2014).

Several caffeine targets sit within or adjacent to the muscle adaptation network. They include adenosine A_1_and A_2_A receptors, ryanodine receptor 1, Ca²^+^ linked kinases, AMP-activated protein kinase (AMPK), and downstream transcriptional regulators such as peroxisome proliferator-activated receptor gamma coactivator 1-alpha (PGC-1α) (Fredholm et al., 2011; Rossi and Dirksen, 2006; Egan and Zierath, 2013). Their presence in the network does not establish that routine oral caffeine dosing meaningfully alters adaptation in humans. Whether caffeine acts as an amplifier, a partial mimic, an attenuator, or only a performance mediator is an empirical question.

The available evidence is asymmetric. Acute caffeine physiology rests on human performance trials, metabolic studies, electrophysiology, and muscle function experiments. Chronic human evidence remains short in duration, narrow in training mode, and largely without biopsy level endpoints. Preclinical studies provide finer mechanistic detail, yet they rely on diverse cell systems, isolated muscles, injury models, and animal training protocols. The concentrations and exposure patterns used in such models often fail to map cleanly onto routine human ergogenic dosing.

This review separates the acute ergogenic question from the chronic adaptation question. We first define caffeine targets that are plausible at concentrations achievable through human oral dosing. We then rank acute human mechanisms by evidential strength, separate chronic human training evidence from training quality mediator evidence, and use three working models to clarify how future studies could distinguish direct signalling effects from performance-mediated changes in training stimulus. We close by integrating preclinical mechanistic evidence and identifying moderators, confounders, and research priorities.

Within this overall framework, we organise discussion around four nested sub-questions, addressed in hierarchical order: (1) whether caffeine directly modifies skeletal muscle molecular signalling; (2) whether caffeine improves chronic training adaptation by enhancing training quality; (3) whether caffeine may impair recovery and thereby attenuate adaptation; and (4) how these mechanisms may coexist depending on dose, timing, exercise modality, and individual characteristics. The central distinction throughout is whether caffeine alters adaptation by directly modifying muscle signalling, or indirectly by increasing the training stimulus delivered to muscle. Without the distinction, a performance advantage during training can be misread as evidence of altered remodelling biology.

To support transparent interpretation, we organize the available evidence into three tiers. Tier 1 direct human training evidence comprises resistance training, blood-flow restriction (BFR) training, sprint interval training, and endurance training, where available. Tier 2 indirect human mechanistic evidence comprises acute exercise studies, low-glycogen or low-carbohydrate trials, and training-quality mediator studies. Tier 3 preclinical mechanistic evidence comprises cell, isolated muscle, and rodent studies. Conclusions are framed accordingly: only Tier 1 trials can speak directly to durable tissue remodelling, whereas Tiers 2 and 3 inform plausible mechanisms but cannot independently establish chronic adaptation. We note two cautions about this scheme. First, blood-flow restriction training is placed within Tier 1 only for evidential ranking; mechanistically, it differs from conventional resistance and endurance training, because vascular occlusion imposes local hypoxia, metabolite accumulation, and augmented motor-unit recruitment (Yin et al., 2025), so we interpret its adaptations as a distinct subcategory rather than pooling them with other modalities. Second, Tier 3 animal and cell evidence is treated as hypothesis-generating: the caffeine concentrations, exposure durations, and metabolic contexts of these models frequently diverge from human training, and the translational caveats detailed in Section 4.3 apply whenever such findings are extrapolated to human chronic adaptation.

## Caffeine pharmacology and adaptation signalling

### Dose translation from cell systems to contracting human muscle

**Figure 1:**
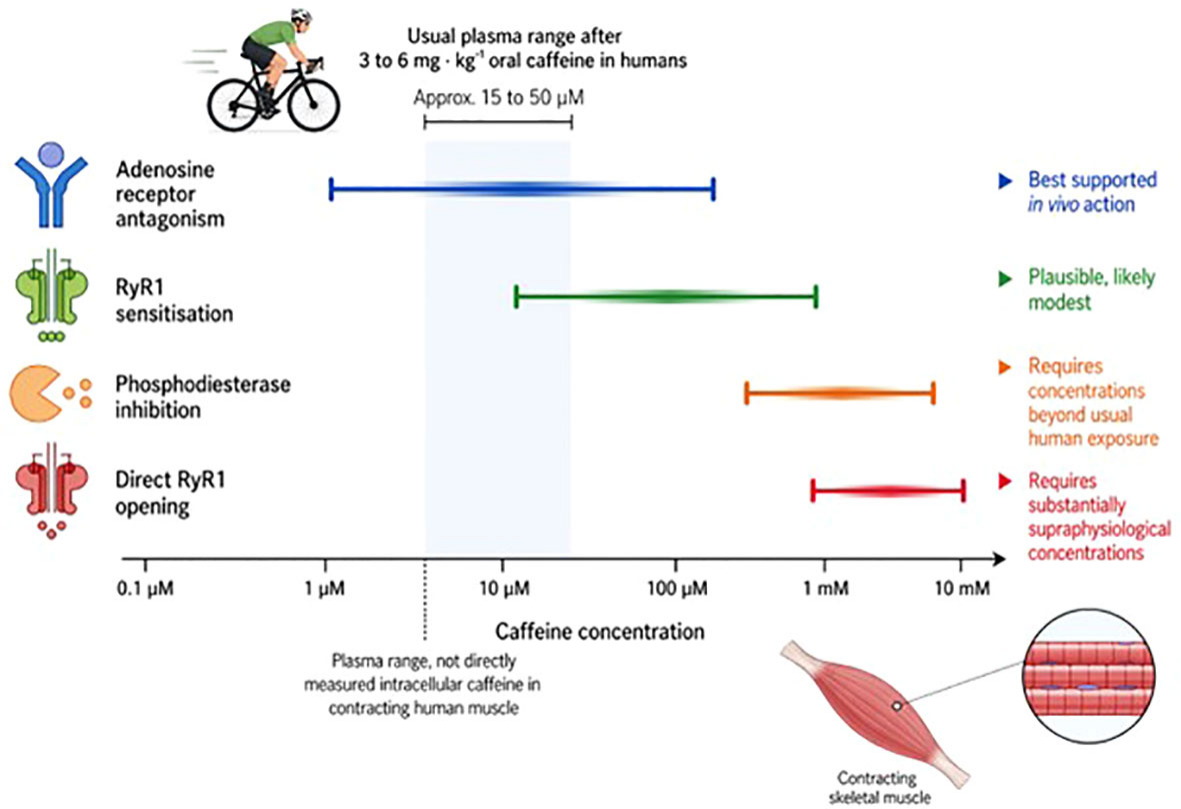
Approximate concentration ranges at which the principal molecular targets of caffeine are engaged, relative to concentrations achieved after oral dosing in humans. The shaded vertical band indicates the usual plasma range after 3 to 6 mg·kg^−^¹. It is not a direct map of intracellular caffeine concentration in contracting human muscle. The figure is intended as a plausibility map rather than a concentration map at each molecular target.

Mechanistic interpretation should begin with dose. **Figure 1** illustrates the approximate concentration range at which the principal molecular targets of caffeine are engaged. Oral intake of 3 to 6 mg·kg^−^¹ usually produces peak plasma concentrations of about 20 to 80 µM within 45 to 90 min. The elimination half-life is 4 to 6 h in many adults (Graham and Spriet, 1995; Magkos and Kavouras, 2005). This 3 to 6 mg·kg^−^¹ band is the consensus ergogenic dose and represents the primary mode and level of intake in the human literature (Guest et al., 2021); the resulting plasma concentrations of about 20 to 80 µM are sufficient to antagonise adenosine A_1_and A_2_A receptors but generally remain below the threshold required for direct mass Ca²^+^ release through RyR1 or for phosphodiesterase inhibition, which is why the most reproducible human effects are receptor-mediated rather than dependent on these higher-concentration targets. Cell and isolated tissue studies often apply millimolar caffeine because such concentrations evoke robust sarcoplasmic reticulum Ca²^+^ release and serve as useful experimental probes (Rousseau et al., 1988; Allen et al., 2008). The gap is not a technical detail. It determines whether an observed effect is likely to occur during routine human supplementation or should be regarded as a pharmacological probe.

Plasma, interstitial, and intracellular concentrations should not be treated as equivalent. Intracellular caffeine in exercising human muscle has not been quantified with the resolution required for precise target mapping. We therefore use 10 to 100 µM as a working exposure range relevant to humans. Within this range, adenosine receptor antagonism is the best supported pharmacological action. RyR1 sensitisation is plausible but probably modest. Phosphodiesterase inhibition and direct RyR1 opening require concentrations well above usual human exposure. Any AMPK effect under routine dosing is more likely to be indirect, operating through altered Ca²^+^ handling or energetic state rather than direct pharmacological activation (Fredholm et al., 1999; Spriet, 2014).

### RyR1 and sarcoplasmic reticulum Ca²^+^ release

RyR1 is the sarcoplasmic reticulum Ca²^+^ release channel that couples T tubule depolarisation to contraction in mammalian skeletal muscle (Rossi and Dirksen, 2006). Caffeine acts as a positive allosteric modulator of RyR1. At high concentrations the compound can evoke the large Ca²^+^ release familiar from contracture experiments. At concentrations closer to those reached after routine oral dosing, the more plausible effect is a modest reduction in the Ca²^+^ threshold for release during ongoing activation (Rousseau et al., 1988; Tallis et al., 2015). Contractile data relevant to humans support that interpretation. Caffeine can potentiate low frequency force and alter Ca²^+^ related contractile behaviour without producing a large change in maximal tetanic force (Tarnopolsky and Cupido, 2000; Tallis et al., 2015; Fukutani et al., 2022).

This distinction matters for adaptation. Ca²^+^ sensitive kinases respond to the timing, amplitude, and frequency of Ca²^+^ transients rather than to a simple threshold event. A modest caffeine induced bias in Ca²^+^ handling could in principle influence CaMK (Ca²^+^/calmodulin-dependent protein kinase) or CaMKKβ signalling (Chin, 2005; Wright et al., 2007). Whether the effect occurs to a meaningful extent in exercising human muscle under oral dosing remains unresolved. At present, the strongest support for the pathway comes from cell and rodent studies rather than direct biopsy evidence in human training studies.

### Adenosine A_1_and A_2_A receptor antagonism

At concentrations achieved after ergogenic oral doses (3 to 6 mg·kg^−^¹), caffeine acts as a non-selective competitive antagonist of adenosine A_1_and A_2_A receptors (Fredholm et al., 1999, 2011). This is the most defensible primary action under routine human dosing. The effects are distributed across the central nervous system, vasculature, endocrine responses, and possibly skeletal muscle. Centrally, adenosine receptor blockade reduces perceived effort and modifies central fatigue during exercise. The mechanism probably explains a large part of the acute ergogenic response (Davis et al., 2003; Meeusen et al., 2013). Peripherally, adenosine receptor antagonism can affect vascular tone, catecholamine release, and metabolic responses (Graham, 2001; Fredholm et al., 2011).

Evidence for adenosine receptor antagonism as a direct skeletal muscle remodelling mechanism is weaker. Repeated receptor blockade may plausibly influence training tolerance, sleep, perceived effort, or recovery behaviour. It is less clear that the same blockade directly shifts mitochondrial biogenesis, protein synthesis, or fibre phenotype in human muscle. The pathway should therefore be separated into a central performance route, which is well supported, and a direct muscle adaptation route, which is not yet well tested.

### Phosphodiesterase inhibition and other low affinity targets

Phosphodiesterase inhibition by caffeine requires concentrations that exceed those achieved with tolerable oral intake (here taken as up to approximately 3 to 6 mg·kg^−^¹ per dose, or about 400 mg per day for a typical adult) (Fredholm et al., 1999; Magkos and Kavouras, 2005). Similar dose problems apply to several other low affinity targets, including direct effects on glycogen phosphorylase and some GABA related mechanisms. Such actions may be useful *in vitro*, but they should not serve as primary explanations for routine caffeine effects in exercising humans. A dose resolution rule follows. Mechanistic claims about humans should be anchored to exposure levels plausible after oral dosing, whereas effects requiring much higher concentrations should be described as experimental probes.

### The Ca²^+^ and CaMK axis

Repeated Ca²^+^ transients during contraction activate CaMK family signalling, including CaMKII (Ca²^+^/calmodulin-dependent protein kinase II) and the upstream CaMKKβ pathway (Rose and Hargreaves, 2003; Chin, 2005). CaMKII phosphorylates class II histone deacetylases, derepresses MEF2 (myocyte enhancer factor 2) dependent transcription, and contributes to the early transcriptional response to contraction. PGC 1α mRNA often rises transiently within hours of exercise and precedes later changes in protein abundance or mitochondrial phenotype (Pilegaard et al., 2003; Wright et al., 2007).

Cell studies show that raising intracellular Ca²^+^ can reproduce parts of the exercise transcriptional response, including mitochondrial biogenic markers (Ojuka et al., 2003). The finding provides a mechanistic basis for considering caffeine as a partial mimic of selected contraction signals. It does not establish that routine human dosing generates the same signal amplitude, timing, or adaptive consequence in trained skeletal muscle.

### The energetic stress and AMPK axis

AMPK is a heterotrimeric energy sensor activated by changes in adenine nucleotide status during contraction and, in some contexts, by CaMKKβ in response to Ca²^+^ signals (Wojtaszewski et al., 2000; Hardie et al., 2012). Human exercise activates AMPK in an intensity dependent and isoform specific manner. Higher intensity exercise drives stronger α2 activation, whereas rodent caffeine studies often show preferential α1 activation (Wojtaszewski et al., 2000; Egawa et al., 2011).

This isoform distinction is important. It argues against a simple equivalence between caffeine and exercise. Caffeine can engage parts of the AMPK network in rodent muscle, but the activation pattern may not match contraction *in vivo* (Egawa et al., 2011; Jensen et al., 2007). Downstream, AMPK can phosphorylate regulators of substrate metabolism and PGC 1α, and it can regulate autophagy and mitophagy through ULK1 (Unc-51-like autophagy-activating kinase 1) dependent mechanisms (Jäger et al., 2007; Laker et al., 2017; Mukai et al., 2014). The adaptive output may therefore depend not only on whether AMPK is activated. It may also depend on which isoform is engaged (Ruas et al., 2012), how long activation persists, and how the signal couples to contraction and recovery.

### PGC 1α and mitochondrial quality control

PGC 1α serves as a useful integrative node, but it should not be treated as a standalone proxy for adaptation. Transient changes in PGC 1α mRNA, phosphorylation, or acetylation do not demonstrate completed mitochondrial remodelling. Training induced mitochondrial adaptation involves biogenesis, dynamics, selective mitophagy, respiratory function, mitochondrial protein import, and proteostatic regulation (Hood, 2019; Memme et al., 2021; Picca et al., 2023).

The classical model links CaMK, AMPK, p38 MAPK (p38 mitogen-activated protein kinase), PGC 1α transcription, and SIRT1 (sirtuin 1) related deacetylation to endurance type adaptation (Handschin and Spiegelman, 2008; Cantó et al., 2009; Philp et al., 2011). The architecture is now broader. Mitochondrial quality control and mitophagy are not secondary background processes but constitutive elements of the adaptive response to repeated contractile stress. Such breadth makes prediction difficult. A partial pharmacological perturbation may shift an early signalling marker without producing a durable change in mitochondrial content or function.

### Plausible points of caffeine entry into the adaptation network

**Figure 2:**
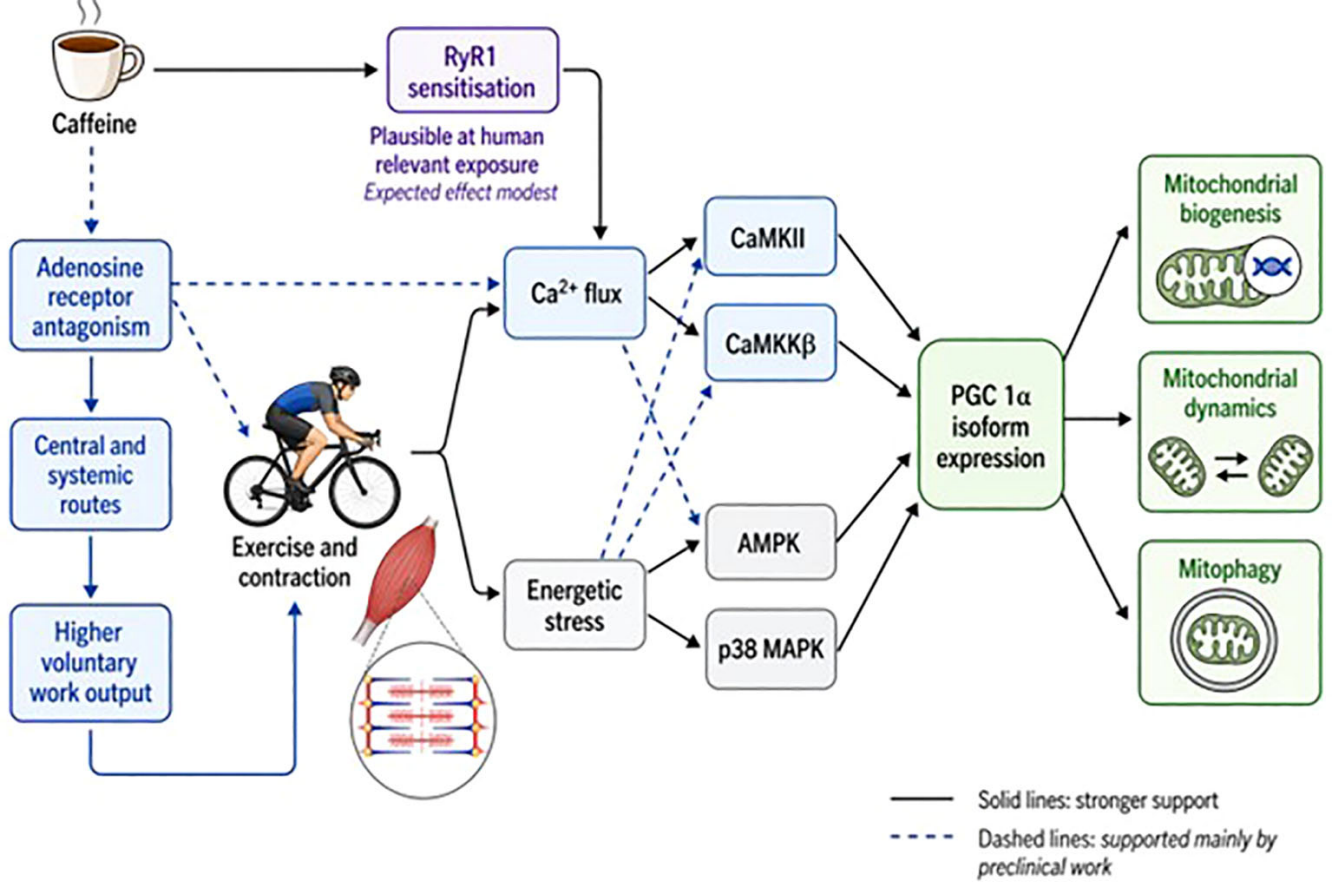
Skeletal muscle signalling network relevant to training adaptation and likely points of caffeine interaction. Contraction related Ca²^+^ flux and energetic stress converge on CaMKII, CaMKKβ, AMPK, and p38 MAPK, influencing PGC 1α isoform expression and downstream programmes of mitochondrial biogenesis, dynamics, and mitophagy. Dashed lines indicate links supported mainly by preclinical work.

**Figure 2** illustrates the skeletal muscle signalling network associated with training adaptation, together with potential points of caffeine interaction. Three caffeine entry points are defensible at human-relevant exposure. The first is modest RyR1 sensitisation, which could bias the Ca²^+^ signal and engage CaMK, CaMKKβ, and AMPK-linked pathways. The second is adenosine receptor antagonism, whose direct muscle role is unclear but whose central and systemic effects are established. The third is indirect. By reducing perceived effort and increasing voluntary output, caffeine can raise the mechanical and metabolic stimulus delivered to the muscle. The training quality pathway is the best supported route in humans because acute changes in perception and output are documented more consistently than direct muscle signalling changes.

## Acute ergogenic mechanisms in humans

The acute human literature is the strongest empirical anchor for caffeine physiology. It includes performance trials, substrate and ion handling studies, electrophysiology, and isolated muscle or fibre experiments. The literature supports a mechanism hierarchy rather than a simple list (Grgic et al., 2020; Guest et al., 2021). Two limitations apply. First, participants have been predominantly male. Extrapolation to women is therefore constrained, especially because caffeine clearance can be influenced by oestrogen status, menstrual cycle phase, and hormonal contraception. Second, habitual caffeine intake is often poorly described, although tolerance at behavioural and receptor levels is plausible (Fredholm et al., 1999; Tarnopolsky and Cupido, 2000; Guest et al., 2018).

### Performance effects across modalities

Taken together, the available meta-analytic evidence indicates that caffeine produces small average improvements in exercise performance, although the magnitude varies by performance domain. For endurance time trial performance, corrected estimates indicate improvements in mean power output of 2.92 ± 2.18% with an effect size of 0.22 ± 0.15, and improvements in completion time of 2.26 ± 2.60% with an effect size of 0.28 ± 0.12 (Southward et al., 2018). For resistance exercise outcomes, caffeine has small effects on maximal strength and power, with pooled SMDs of 0.20 for maximal strength, 0.21 for upper body strength, 0.15 for lower body strength, and 0.17 for muscle power (Grgic et al., 2018). Earlier evidence also reported small effects on maximal voluntary contraction strength and muscular endurance, with overall effect sizes of 0.19 and 0.28, respectively; however, larger effects were mainly observed for knee extensor strength, approximately 7%, ES = 0.37, and open endpoint muscular endurance tests, approximately 18%, ES = 0.37 (Warren et al., 2010). Therefore, caffeine appears to have a modest ergogenic effect on average, but the response should not be treated as uniform across exercise modes, participant groups, and outcome definitions.

Placebo and expectancy effects also contribute under some conditions (Beedie et al., 2006). The contribution does not undermine the physiological effect. It indicates that the measured performance response combines central pharmacology, perception, motivation, and peripheral physiology. For chronic adaptation, the implication matters. An increase in session output can change the training stimulus even when skeletal muscle signalling is not directly modified by caffeine.

### Substrate metabolism

Early biopsy studies reported lower glycogen use during the first phase of exercise and higher circulating free fatty acids after caffeine ingestion (Costill et al., 1978; Spriet et al., 1992; Chesley et al., 1998). The findings led to a glycogen sparing hypothesis. Later work under more controlled conditions narrowed that interpretation. Caffeine did not consistently alter net glycogen use or fuel selection within exercising human skeletal muscle, despite expected systemic changes in catecholamines and circulating substrates (Graham et al., 2000; Hulston and Jeukendrup, 2008).

The current interpretation should distinguish systemic substrate availability from direct muscle fuel selection. The latter is not well supported as the primary explanation for acute ergogenicity in trained humans. The substrate pathway remains relevant as a mediator under low glycogen or carbohydrate restriction settings, but it is not sufficient evidence for direct enhancement of adaptation (Bartlett et al., 2015).

### Ion handling and muscle excitability

A better supported peripheral mechanism involves potassium handling. During intense repeated depolarisation, extracellular K^+^ can accumulate faster than it is cleared, contributing to membrane depolarisation and force loss (Clausen, 2003; McKenna et al., 2008). Human studies show that caffeine attenuates increases in plasma and interstitial K^+^ and improves intense intermittent exercise performance under comparable conditions (Lindinger et al., 1993; Mohr et al., 2011).

The mechanism probably involves catecholamine mediated stimulation of Na^+^/K^+^ ATPase activity, with possible additional effects on excitability. The pathway is directly relevant to acute maintenance of force and performance during intense exercise. It is less directly relevant to long term remodelling unless the maintained output increases the repeated training stimulus.

### Contractile function

Contractile data provide a separate peripheral line of evidence. In the primary human study cited here, oral caffeine ingestion at 6 mg·kg^−^¹ potentiated electrically evoked low frequency force during 20 Hz stimulation, whereas this effect was not observed during 40 Hz stimulation or for maximal voluntary contraction strength (Tarnopolsky and Cupido, 2000). Skinned and isolated fibre work supports the interpretation that caffeine affects Ca²^+^ handling more than cross bridge function, although concentration dependence is central (Rousseau et al., 1988; Fukutani et al., 2022).

The whole body performance contribution of the pathway is difficult to quantify. Altered contractile behaviour can reasonably be treated as a contributory mechanism in selected contexts, especially tasks involving repeated submaximal contractions (Tallis et al., 2015; Black et al., 2015). It should not be ranked above central adenosine receptor antagonism or the training quality pathway in the general hierarchy.

### Intracellular signalling in human muscle

The acute signalling question remains less resolved. Exercise itself strongly activates Ca²^+^ linked kinases and AMPK, with intensity and fibre recruitment shaping the response (Wojtaszewski et al., 2000; Egan and Zierath, 2013). Any caffeine effect during exercise is therefore superimposed on an already strong contraction signal. Human biopsy studies specifically designed to test caffeine effects on canonical adaptation signalling are sparse. The sparseness is not strong negative evidence. It indicates that the relevant human mechanistic studies have not been conducted with the necessary density of sampling and endpoints.

### Ranked interpretation of acute mechanisms

**Table 1** summarises acute mechanisms of action of caffeine in humans. The acute human hierarchy is restrained. Central adenosine receptor antagonism contributes substantially to the aggregate ergogenic effect. K^+^ handling has direct human support during intense intermittent exercise. Ca²^+^ related contractile effects are plausible but smaller and more context dependent. Direct muscle fuel selection changes and robust caffeine specific biopsy level signalling amplification remain weakly supported. The hierarchy explains acute performance better than chronic adaptation. A pathway that changes performance within minutes does not automatically change mitochondrial content, protein turnover, or tissue structure over weeks.

**Table 1:** Acute caffeine mechanisms in humans, classified by site, evidence strength, dose plausibility, relevance to chronic adaptation, and main limitation.

Mechanism	Primary site	Acute human evidence	Dose plausibility	Relevance to chronic adaptation	Main limitation
Adenosine receptor antagonism	Adenosine A_1_and A_2_A receptors, mainly central nervous system with systemic contributions	Strong. Reproducible reductions in perceived effort and increases in voluntary output across several exercise modes.	High at 3 to 6 mg·kg^−^¹. Plasma concentrations are compatible with receptor antagonism.	Indirect. Most likely acts through session quality and tolerable work output.	No direct human evidence that chronic receptor antagonism changes muscle remodelling biology.
Potassium handling	Na^+^/K^+^ ATPase activity and extracellular K^+^ regulation in contracting muscle	Moderate. Direct human support in intense intermittent exercise.	Plausible at routine ergogenic doses.	Low to indirect. Relevant mainly if maintained excitability increases repeated training work.	Strongest evidence is during the exercise bout, not during recovery or remodelling.
RyR1 and Ca²^+^ handling	Ryanodine receptor 1 and sarcoplasmic reticulum Ca²^+^ release	Moderate. Low frequency force potentiation and Ca²^+^ related contractile effects in human muscle.	Concentration sensitive. Sensitisation plausible in the human exposure range; large release requires millimolar caffeine.	Possible. A biased Ca²^+^ signal could engage CaMK and CaMKKβ arms.	No chronic human trial links caffeine related Ca²^+^ sensitisation to tissue endpoints.
Substrate metabolism	Catecholamines, adipose lipolysis, hepatic glucose output, exercising muscle substrate use	Limited as an independent mechanism. Early glycogen sparing is not consistently reproduced.	Systemic effects plausible. Direct muscle fuel selection changes are inconsistent.	Low to indirect. Most relevant to preservation of work output in low carbohydrate settings.	Difficult to separate caffeine effects from the broader catecholamine response to exercise.

RyR1, ryanodine receptor 1; CaMK, Ca²^+^/calmodulin-dependent protein kinase; CaMKKβ, Ca²^+^/calmodulin-dependent protein kinase kinase β; Na^+^/K^+^ ATPase, sodium-potassium adenosine triphosphatase.

## Chronic adaptation evidence and working models

### Direct human training data

The available human training literature should be separated into related but not equivalent evidence categories. (i) Direct training trials test whether pre-session caffeine alters performance or strength outcomes during short conventional resistance training blocks (Giráldez Costas et al., 2021; Tamilio et al., 2021). (ii) Mixed sport performance training studies, including low-load blood-flow restriction work, are more informative for neuromuscular control than for tissue remodelling (Lin et al., 2025). (iii) Acute training-quality mediator studies, encompassing low-glycogen or low-carbohydrate trials and endurance and interval mediator work, address how caffeine modifies session output rather than chronic adaptation (Lane et al., 2013; Soegaard et al., 2025). Only true repeated training interventions can address adaptation; acute studies under substrate restriction mainly address training-quality mediation and should not be pooled as evidence for one adaptive phenotype.

Direct human training data remain limited and do not support a uniform additive effect of caffeine on strength adaptation. In a 4 week bench press training study, ingestion of caffeine at 3 mg·kg^−^¹ before each training session did not meaningfully enhance 1RM gains compared with placebo, with increases of 13.5 ± 7.8% and 11.3 ± 5.3%, respectively, p = 0.53; however, the caffeine group showed training related improvements in mean velocity, peak velocity, mean power, and peak power across a wider range of relative loads (Giráldez Costas et al., 2021). In rugby union players, caffeine ingestion at 3 mg·kg^−^¹ before training did not augment 1RM gains across six resistance exercises, with percentage increases of 16% vs 16% for chest press, 19% vs 22% for seated shoulder press, 24% vs 25% for squat, 24% vs 24% for deadlift, 42% vs 40% for hang clean, and 30% vs 27% for power clean in the placebo and caffeine groups, respectively; although repetitions to failure were slightly higher with caffeine for several exercises, these differences were small (Tamilio et al., 2021). More recently, in a 4 week low load BFR wrist extensor training study, caffeine at 6 mg·kg^−^¹ did not significantly enhance MVC gains compared with BFR alone, 25.7 ± 6.3% vs 20.5 ± 5.4%, but improved force release control, including a reduction in ramp down force fluctuation RMS from 12.28 ± 0.54 to 11.32 ± 0.35%MVC/s and an increase in SampEn from 0.483 ± 0.023 to 0.559 ± 0.023 (Lin et al., 2025). These studies indicate that repeated pre exercise caffeine ingestion may influence selected performance or neuromuscular control outcomes, but current evidence does not show consistent additional gains in maximal strength.

Across these trials, three limitations are consistent: absence of biopsy-level mechanistic endpoints, limited ability to distinguish retained adaptation from acute caffeine responsiveness, and short intervention duration. The studies therefore test whether caffeine changes performance-related outcomes during training, not whether it directly modifies skeletal muscle remodelling.

### Endurance and interval training related evidence

The endurance related evidence base (**Table 2**) is now broader than the resistance training literature alone, but it still does not resolve tissue adaptation. Interpreted under the three working models (H1 amplifier, H2 partial mimic with training-quality mediation, H3 attenuator), these studies are most compatible with H2; none measured biopsy-level endpoints and therefore none can refute either H1 or H3. Zhao and Liu examined a twelve week sprint interval training intervention in overweight and obese women. Participants consumed 3 mg·kg^−^¹ caffeine before training sessions. The caffeine condition was associated with greater reductions in fat mass and greater gains in strength, cardiorespiratory fitness, fasting glucose, and adipokine outcomes. The study is useful because it is a true training intervention with an interval modality. The limits are equally clear. The sample was small, the population specific, and muscle biopsy endpoints were not included.

**Table 2:** Human studies relevant to repeated caffeine exposure, endurance or interval training, and training quality mediation.

Study	Exercise context	Participants	Exposure	Endpoint level	Mechanistic endpoints	Main inference
Giráldez Costas et al., 2021	Bench press resistance training for four weeks	Recreationally trained men	3 mg·kg^−^¹ before sessions, placebo controlled	Strength, movement velocity, power	None. No biopsy or molecular markers.	Velocity and power related gains were larger with caffeine, while maximal strength was similar. Cannot test tissue remodelling.
Tamilio et al., 2021	Multi exercise resistance training for seven weeks	Male rugby union players	3 mg·kg^−^¹ before two weekly sessions, placebo controlled	Maximal strength and training volume	None. No biopsy or molecular markers.	Training volume was modestly higher with caffeine, but maximal strength was unchanged and acute responsiveness attenuated.
Lin et al., 2025	Low load resistance exercise with blood flow restriction	Untrained participants	Pre session caffeine, placebo controlled	Strength, force release precision, motor unit discharge variability	Surface electromyography and force steadiness only.	Most informative for neuromuscular control. It does not test hypertrophic or mitochondrial remodelling.
Zhao and Liu, 2025	Sprint interval training for twelve weeks	Overweight and obese women	3 mg·kg^−^¹ before sessions, placebo controlled	Fat mass, lower body strength, cardiorespiratory fitness, fasting glucose, adipokines	Circulating metabolic markers. No muscle biopsy.	Closest available interval training evidence. Suggests improved training outcomes, but tissue mechanism is unresolved.
Wu and Jiang, 2024	Plyometric jump training for six weeks	Male basketball players	3 or 6 mg·kg^−^¹ before sessions, placebo controlled	Jump, sprint, change of direction, strength, Wingate, maximal oxygen uptake	Field and laboratory performance only. No biopsy.	Adds mixed sport and aerobic fitness outcomes, but cannot infer mitochondrial tissue adaptation.
Lane et al., 2013	Endurance interval session under normal or low glycogen	Endurance trained cyclists and triathletes	Acute caffeine crossover	Interval power output	No chronic training endpoint.	Caffeine can preserve session output under low glycogen. This is mediator evidence, not adaptation evidence.
Soegaard et al., 2025	High intensity interval exercise followed by carbohydrate restricted recovery	Female endurance athletes	300 mg caffeine in a crossover design	Fat oxidation and twenty min time trial performance	No chronic training endpoint.	Caffeine may preserve performance during carbohydrate restriction. This informs training quality, not chronic remodelling.

mg·kg^−^¹, milligrams per kilogram of body mass.

The table separates true training interventions from acute mediator studies.

Wu and Jiang examined six weeks of plyometric jump training in male basketball players. Participants received 3 or 6 mg·kg^−^¹ caffeine before sessions. Outcomes included jump, sprint, change of direction, strength, Wingate performance, and maximal oxygen uptake, several of which improved more with caffeine than with placebo, with some evidence of a dose-dependent pattern favouring the higher 6 mg·kg^−^¹ dose (Wu and Jiang, 2024). The study expands the exercise modality beyond conventional resistance training, but the intervention is a mixed sport performance model rather than a direct endurance remodelling study. Again, no muscle biopsy endpoints were included.

Lane and colleagues and Soegaard and colleagues are better classified as endurance mediator studies than as chronic adaptation trials. Lane and colleagues showed that caffeine increased interval power output in trained cyclists and triathletes under both normal and low muscle glycogen conditions. Soegaard and colleagues studied female endurance athletes under carbohydrate restriction after high intensity interval exercise and found that caffeine helped preserve next day performance while carbohydrate restriction increased fat oxidation. Such trials are useful because they show how caffeine may preserve training quality when substrate availability is constrained. They do not show that caffeine enhances mitochondrial adaptation under chronic training conditions.

Interpreted within the three-tier framework introduced in the Introduction and the working models formalised in Section 4.2 (Working models, H1–H3), these endurance and interval studies cannot discriminate among the three hypotheses. None of the trials measured tissue-level remodelling endpoints (mitochondrial respiration, mitochondrial content, protein synthesis, or mitophagy), and none included caffeine-abstinent post-training testing. The Zhao and Liu sprint interval trial is the closest Tier 1 design and is broadly compatible with H2 (partial mimic with training-quality mediation), in which caffeine modestly amplifies session output without a demonstrated tissue-level divergence. The Wu and Jiang plyometric trial likewise reports performance gains consistent with H2 rather than with H1 (amplification of remodelling under matched external work). The Lane et al. and Soegaard et al. studies are Tier 2 mediator evidence and speak to training-quality preservation under substrate restriction rather than to durable adaptation; they neither support nor refute H1 or H3.

Testable predictions follow directly from this framing. H1 predicts a between-group difference in mitochondrial respiration or content under work-matched endurance training. H2 predicts session-output and acute signalling gains without a clear separation in core tissue endpoints, which is the pattern most compatible with the available data. H3 predicts dissociation between preserved acute performance and reduced recovery-phase or biopsy-level outcomes, including possible attenuation of post-exercise protein synthesis or mitophagy markers, and would require objective sleep monitoring and caffeine-abstinent post-test biopsies to detect. Until at least one Tier 1 trial incorporates these endpoints, the endurance and interval evidence base should be read as performance-mediator data, not as evidence for or against direct modification of skeletal muscle adaptation.

### Preclinical evidence and direct mechanisms

When preclinical findings are used to support broader interpretations, four sources of translational uncertainty should be made explicit: (i) caffeine concentrations applied in cell and rodent work frequently exceed those achievable through tolerable human oral intake (here, intake at consensus ergogenic and safety levels, namely up to approximately 3 to 6 mg·kg^−^¹ per dose or about 400 mg per day for a typical adult; Guest et al., 2021); (ii) exposure durations and dosing patterns often differ substantially from peri-exercise human dosing; (iii) metabolic context, including fed or fasted state, glycogen status, and hormonal milieu, may not match trained human conditions; and (iv) tissue-specific responses observed in rodent fast-twitch dominant muscle or in immortalised muscle cell lines may not generalise to mixed-fibre skeletal muscle in exercising humans.

Preclinical evidence should be stratified by model. Cell systems are useful for testing whether caffeine engages Ca²^+^ linked or AMPK related pathways under controlled exposure. Isolated muscle preparations add information on contractile and metabolic responses. Intact animal studies are more relevant to tissue adaptation, but injury, regeneration, and engineered tissue models do not directly reproduce healthy human training adaptation. **Table 3** summarises the mechanistic and animal experimental evidence relevant to caffeine and skeletal muscle adaptation.

**Table 3:** Mechanistic and animal evidence relevant to caffeine and skeletal muscle adaptation.

Study	Model	Exposure and exercise context	Direct endpoint	Directional signal	Translational limit
Jensen et al., 2007	Isolated rat and mouse soleus	Caffeine exposure without whole body training	Ca²^+^ release, CaMKK, α1 AMPK, glucose uptake	Supports Ca²^+^ linked AMPK activation and glucose uptake.	Isolated muscle exposure. Dose and contraction context differ from human training.
Egawa et al., 2011	Rat skeletal muscle	Caffeine exposure	AMPK isoform activation	Preferential α1 AMPK activation.	Pattern differs from contraction, where α2 is prominent during higher intensity human exercise.
Mathew et al., 2014	Skeletal muscle cells	Caffeine treatment	Autophagy, CaMKKβ, CaMKII, AMPK, LC3b	Supports Ca²^+^ dependent AMPK autophagy pathway.	Cell model and exposure conditions are not direct human dosing equivalents.
Vaughan et al., 2012	Rhabdomyosarcoma cells	Caffeine treatment	PGC 1α expression, mitochondrial content, oxidative metabolism	Supports mitochondrial biogenesis related signalling.	Tumour derived cell line. Not contracting adult human muscle.
Enyart et al., 2020	C2C12 skeletal muscle cells	Low dose caffeine exposure	Fatty acid use and mitochondrial turnover markers	Supports metabolic and mitochondrial turnover effects.	Cell culture endpoint. No repeated contraction or tissue integration.
Moore et al., 2017	Mice and cultured muscle cells	Caffeine with stimulated contraction and growth assessment	AMPK, mTOR pathway proteins, protein synthesis, hypertrophy	Does not support a simple anabolic amplifier model.	Mouse and cell context. Not a human training trial.
da Costa Santos et al., 2011	Wistar rats with low intensity exercise	Chronic caffeine intake and training	Muscle damage markers and inflammatory infiltration in soleus	Suggests altered damage and inflammatory response.	Repair and inflammation endpoints, not direct adaptive phenotype.
Vieira et al., 2017	Rats with high intensity interval training	Caffeine 4 or 8 mg·kg^−^¹ combined with training	Muscle Ca²^+^ ATPase, AChE, glycogen, left ventricular thickness	Caffeine prevented selected training induced changes.	Animal HIIT model. Cardiac and muscle endpoints mixed.
Vieira et al., 2017	Rats with high intensity exercise	Caffeine combined with training	Antioxidant enzymes and Na^+^/K^+^ ATPase related outcomes	Caffeine blocked selected exercise induced stress adaptations.	Main endpoints include brain and behavioural measures as well as enzyme activity.
Esteca et al., 2024	Mouse skeletal muscle regeneration with Parkin manipulation	Caffeine during regeneration after injury	Mitochondrial quality control, mitochondrial capacity, Parkin dependence	Supports a direct role in mitochondrial quality control during regeneration.	Regeneration biology differs from training adaptation in non injured muscle.
Steffen et al., 2025	C2C12 cells, tendon cells, engineered ligaments, mice with wheel running	Chronic caffeine exposure with tissue and exercise models	Protein synthesis, collagen, mechanical strength, muscle mass adaptation	Supports an attenuation signal for protein synthesis and selected tissue adaptation.	Single recent study. Requires independent replication and human dose resolved testing.

AMPK, AMP-activated protein kinase; CaMKK, Ca²^+^/calmodulin-dependent protein kinase kinase; CaMKKβ, CaMKK β-isoform; CaMKII, Ca²^+^/calmodulin-dependent protein kinase II; PGC-1α, peroxisome proliferator-activated receptor gamma coactivator 1-alpha; LC3b, microtubule-associated protein 1 light chain 3 beta; mTOR, mechanistic target of rapamycin; AChE, acetylcholinesterase; Na^+^/K^+^ ATPase, sodium-potassium adenosine triphosphatase; HIIT, high-intensity interval training.

The table separates pathway evidence from direct human remodelling evidence.

Several mechanistic findings recur. In rodent soleus, caffeine induced sarcoplasmic reticulum Ca²^+^ release can activate CaMKK linked α1 AMPK and increase glucose uptake (Jensen et al., 2007). In skeletal muscle cells, caffeine can promote autophagy through Ca²^+^ dependent AMPK activation involving CaMKKβ and CaMKII (Mathew et al., 2014). Other cell work links caffeine to PGC 1α expression, mitochondrial content, fatty acid use, and mitochondrial turnover (Vaughan et al., 2012; Enyart et al., 2020). The findings support the partial mimic model, but they do not establish human training adaptation.

Animal and tissue model data are directionally mixed. Some findings support mitochondrial quality control and regeneration pathways, including a Parkin dependent role during skeletal muscle regeneration (Esteca et al., 2024). Other studies suggest attenuation or interference. In rats, caffeine prevented selected enzyme and cardiac structural changes induced by high intensity interval training, including muscle Ca²^+^ ATPase and AChE related outcomes (Vieira et al., 2017). In a separate high intensity exercise rat model, caffeine blocked selected antioxidant and Na^+^/K^+^ ATPase changes (Vieira et al., 2017). Moore and colleagues found that caffeine did not enhance contraction associated anabolic signalling or hypertrophy in mice. Steffen and colleagues reported reduced protein synthesis signals, lower engineered ligament strength, and attenuation of exercise induced muscle mass gains in mice under chronic caffeine exposure. The field therefore contains both mimetic and attenuation signals.

### Emerging attenuation signal

The attenuation hypothesis should be considered without overinterpreting the evidence. Steffen and colleagues reported that chronic caffeine exposure reduced protein synthesis in muscle and tendon related models. Engineered ligament mechanical properties were lowered, and exercise associated muscle mass gains were limited in mice. Because this finding comes from a recent single study (Steffen et al., 2025), it should be treated as hypothesis generating until replicated. Its value lies in defining a biologically plausible direction of effect that earlier human performance studies were not designed to detect.

The attenuation model does not require that caffeine impair acute performance. A compound could preserve or improve session output while reducing selected recovery phase processes. The antioxidant literature provides a formal analogy rather than a direct mechanism. Vitamins C and E can attenuate some molecular adaptations to endurance training without necessarily producing a large immediate performance penalty (Gomez Cabrera et al., 2008; Ristow et al., 2009; Paulsen et al., 2014). The analogy supports only the causal principle that exogenous modification of exercise related signals can sometimes reduce adaptation. It does not imply that caffeine acts through redox mechanisms.

### Three working models and evidential tests

**Figure 3:**
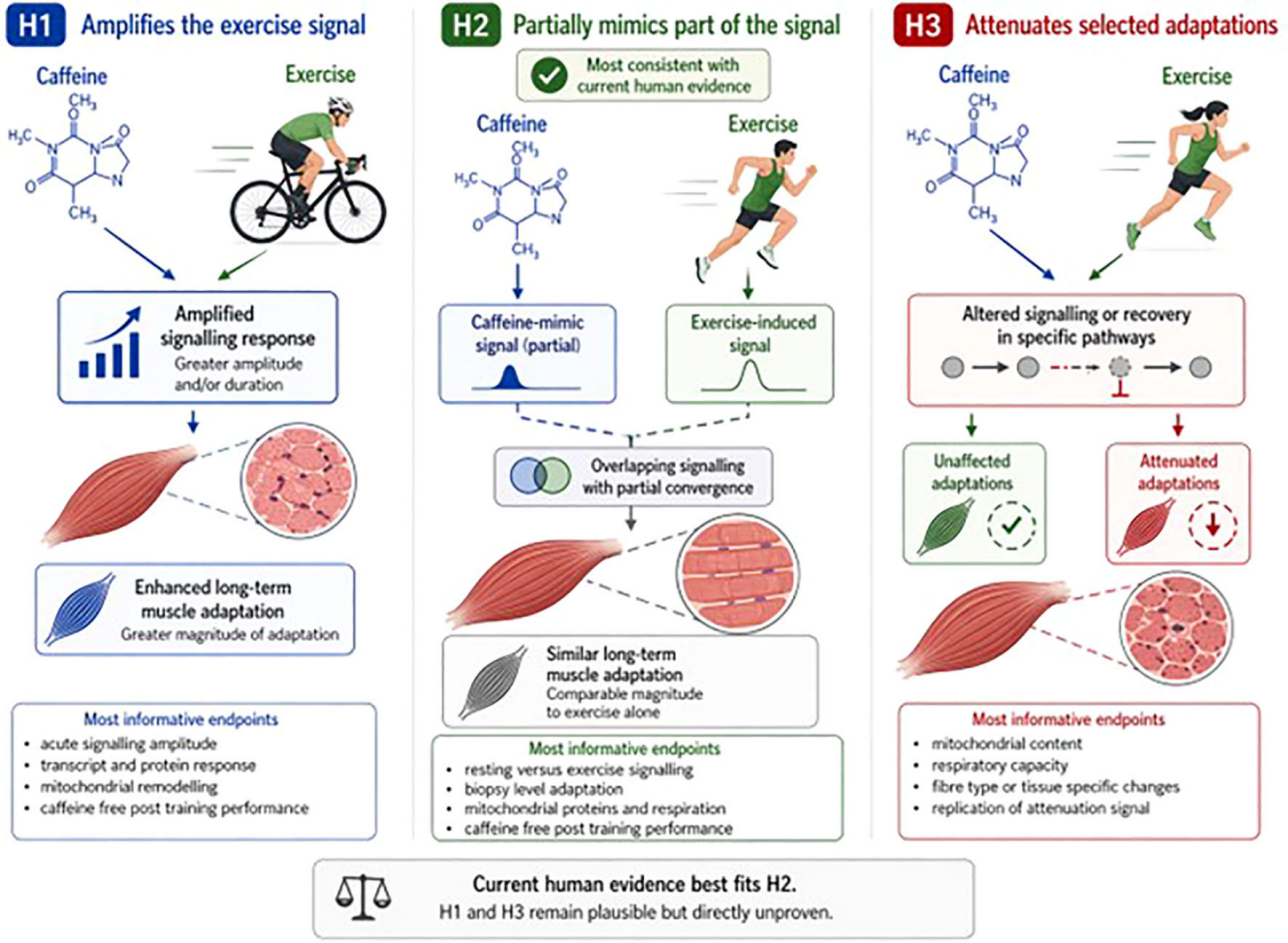
Three working models relating caffeine exposure to skeletal muscle adaptation. H1 predicts amplification when work is matched. H2 predicts overlap with selected acute signals but limited durable tissue divergence. H3 predicts attenuation of selected recovery or tissue processes. Current human evidence best fits H2, while H1 and H3 remain unresolved.

Three concepts should be separated (**Figure 3**). A mimic reproduces part of the exercise signal without providing an equivalent training stimulus. An amplifier increases the adaptive response to a matched training stimulus. A performance mediator increases the stimulus itself by allowing more work to be completed.

Under H1, caffeine acts as an amplifier. The model predicts superior tissue remodelling endpoints even when external work is matched across groups. Examples would include higher mitochondrial respiration, greater mitochondrial content, stronger signalling induced by contraction after standardised exercise, or greater protein synthesis after the same mechanical stimulus. Human evidence does not yet demonstrate the model.

Under H2, caffeine acts as a partial mimic. It overlaps with part of the contraction signal through Ca²^+^ handling, CaMKKβ, AMPK, or PGC 1α related pathways. The added input is not large enough to produce consistent long term divergence once exercise already provides a strong stimulus. The model predicts modest changes in session performance or acute signalling without clear separation in core tissue endpoints. Current human evidence is most compatible with this model.

Under H3, caffeine acts as an attenuator. Repeated exposure reduces selected recovery or tissue processes through sleep mediated recovery impairment, receptor adaptation, altered translational biology, or tissue specific effects on tendon and muscle. The model predicts dissociation between preserved acute session output and reduced recovery phase or tissue adaptation endpoints. It remains plausible but unproven in humans.

### Training quality mediation versus direct signalling

**Figure 4:**
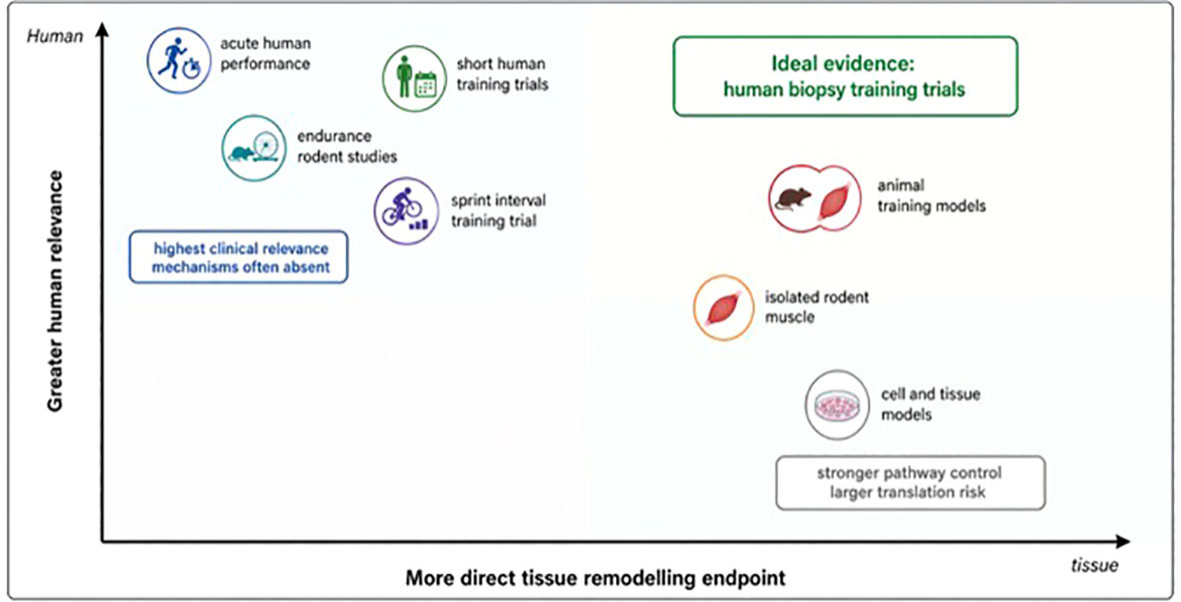
Evidence map across levels of inference. Acute human performance evidence has high relevance but usually lacks tissue endpoints. Cell, isolated muscle, animal training, and engineered tissue models have stronger pathway control but larger translation risk. The missing evidence space is a human training trial with biopsy level endpoints.

The current human literature does not establish whether repeated caffeine exposure changes mitochondrial respiration, mitochondrial content, mitophagy, protein synthesis, fibre type distribution, capillarisation, or structural remodelling (**Figure 4**). It also does not establish whether pre session only dosing and continuous daily exposure produce the same biological effect. Human evidence supports acute performance and possible training quality mediation, but it does not assign a consistent direction of effect on tissue level adaptation.

## Moderators, confounders, and research priorities

These models cannot be separated by performance outcomes alone; they require study designs that isolate exposure pattern, external training load, recovery state, and tissue-level endpoints. Several sources of distortion prevent a clean answer to the chronic adaptation question, and they should be framed as design variables required to discriminate the three working models above. They should be treated as design variables rather than background limitations.

### Exposure pattern and habituation

Caffeine consumed before exercise sessions and continuous daily caffeine intake are not equivalent interventions. Pre session dosing produces pulsatile exposure around training. Daily intake produces repeated receptor occupancy, greater scope for tolerance, and greater likelihood of sleep interaction. Most chronic human trials use pre session dosing, yet many practical recommendations implicitly generalise across both patterns. Such generalisation is not justified without direct evidence.

Habitual consumers and low consumers also occupy different pharmacological states. Acute performance responses may attenuate with repeated exposure, and post intervention testing under ongoing caffeine exposure may capture retained acute responsiveness rather than adaptation. Future trials should prespecify exposure as pre session only, daily independent of training, or combined daily plus pre session use. A withdrawal or caffeine abstinent post-test should be included when feasible.

### Recovery and sleep

Adaptation is expressed during recovery. Caffeine can alter sleep duration and architecture depending on dose, timing, and individual sensitivity (Clark and Landolt, 2017; O’Callaghan et al., 2018). Specifically, evening or higher-dose caffeine has been shown to lengthen sleep-onset latency, shorten total sleep time, reduce sleep efficiency, and suppress slow-wave (deep) sleep, with the magnitude of these changes scaling with the dose and its proximity to bedtime (Clark and Landolt, 2017; O’Callaghan et al., 2018). Sleep mediated recovery impairment is therefore a plausible indirect route through which repeated caffeine exposure could reduce selected adaptations, especially when training occurs late in the day. Existing chronic trials rarely measure sleep objectively. For a chronic caffeine training study, sleep monitoring should be considered a core design feature.

### Training mode and adaptive outcome

Endurance and resistance exercise share parts of the signalling network, but their dominant upstream stimuli and adaptive outcomes differ (Egan and Zierath, 2013; Hawley et al., 2014). Evidence from short resistance training cannot be generalised to mitochondrial adaptation after endurance training. Evidence from sprint interval training in overweight or obese women cannot be generalised to trained endurance athletes without qualification. Evidence from regeneration models cannot be treated as equivalent to adaptation in non injured trained muscle. Each training mode requires endpoint matched interpretation.

### Biological sex and hormonal status

Biological sex remains understudied. Many acute and chronic caffeine trials are predominantly male. Sex linked differences in caffeine clearance, oestrogen exposure, menstrual cycle phase, and hormonal contraception can influence plasma caffeine concentration and possibly downstream responsiveness (Tarnopolsky and Cupido, 2000; Guest et al., 2018). Existing chronic trials are not powered to test sex specific effects on muscle adaptation. Future studies that include women should document menstrual cycle phase, hormonal contraception, and test timing, or justify why such factors are unlikely to affect interpretation.

### Genotype

CYP1A2 (cytochrome P450 1A2) genotype can influence caffeine clearance and has been linked to exercise performance responses in some studies, although effect size and reproducibility remain debated (Womack et al., 2012; Guest et al., 2018). ADORA2A (adenosine A2A receptor gene; ADORA1 denotes the adenosine A1 receptor gene) variation can shape alerting and anxiogenic responses and habitual intake patterns (Rogers et al., 2010). Genotype is therefore a plausible moderator of caffeine response, but no chronic human training study has sufficient power to test genotype by exposure effects on muscle remodelling endpoints. Genotyping is not essential for every trial. It should be considered when sample size permits interaction based inference.

### Future recommendations

**Figure 5:**
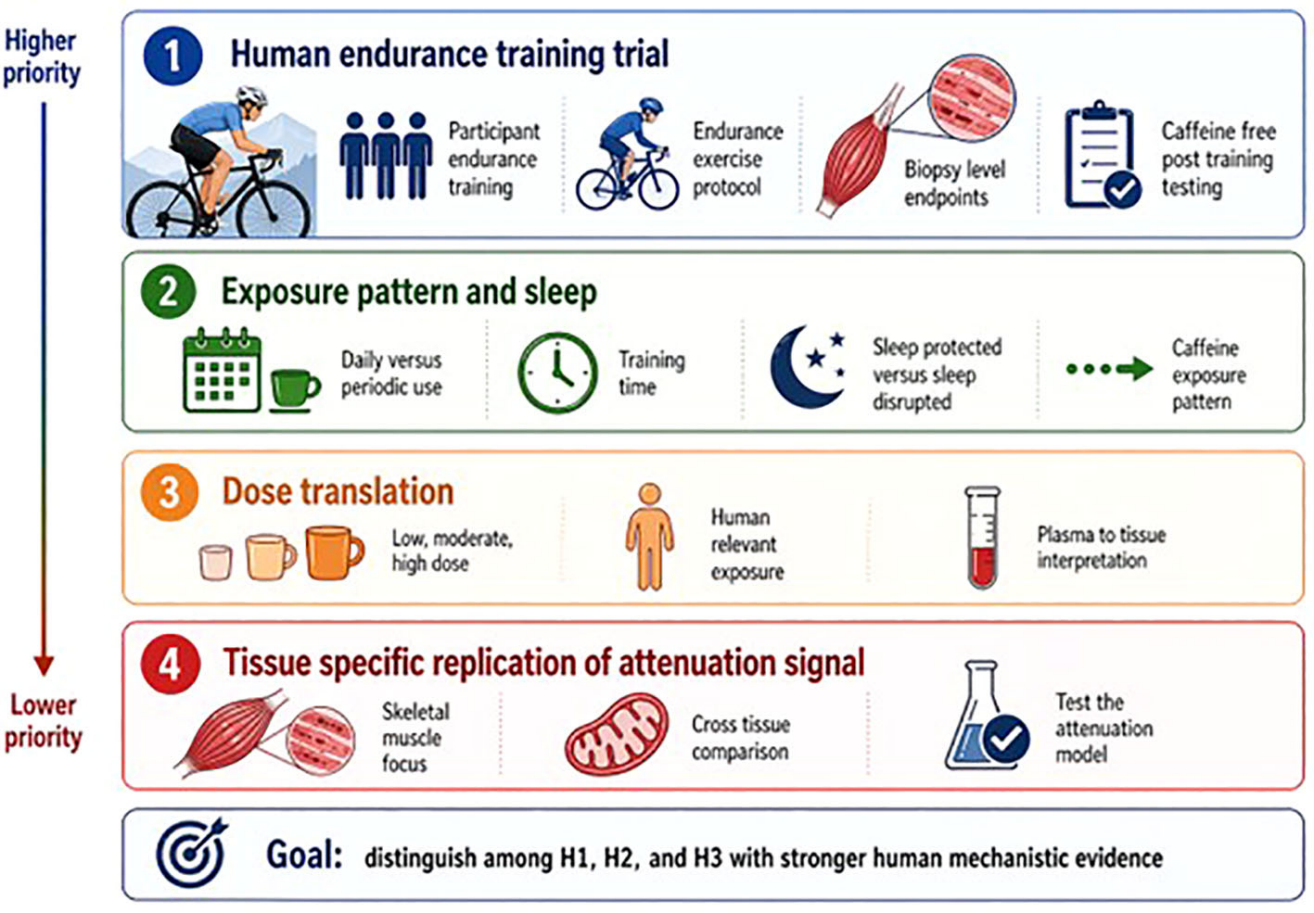
Priority ordering for research designed to discriminate among the three working models. Top priority is a human endurance training trial with biopsy level endpoints and caffeine abstinent post training testing. Other priorities address exposure pattern, sleep, dose translation, and tissue specific replication of attenuation signals.

The first priority is a human endurance training trial with mechanistic endpoints (**Figure 5**). The trial should last at least eight to twelve weeks, include recreationally active to trained participants, and use mitochondrial respiration and mitochondrial content as primary endpoints. Secondary endpoints should include PGC 1α related signalling, AMPK and CaMK pathway markers, protein synthesis or proteomic readouts, and markers of mitophagy such as ULK1 related signalling and mitochondrial lysosomal targeting. Resting biopsies and standardised exercise challenge biopsies should be separated. Post training phenotyping should be performed under caffeine abstinent conditions and, in a separate test, with acute caffeine exposure.

The second priority is direct comparison of exposure patterns. Pre session caffeine, daily caffeine, and combined exposure should not be treated as interchangeable. Objective sleep monitoring, training load recording, and withdrawal testing would allow receptor adaptation and recovery hypotheses to be tested within the same design.

The third priority is dose resolved preclinical work. Experiments should include concentrations close to the human plasma range, use temporal exposure patterns that resemble pre session or daily intake, and capture both contraction and recovery phases. Such design would render animal and cell work more interpretable for human trials.

The fourth priority is replication of attenuation signals. Studies should separate skeletal muscle, tendon, and engineered tissue outcomes, and should test whether attenuation depends on dose, timing, tissue type, and recovery state. The recent attenuation findings are important because they identify a plausible risk model. They should not be translated into strong practice recommendations before replication. Taken together, these four priorities are presented as forward-looking research recommendations rather than as criticisms of individual studies; the corresponding features of the current evidence base, namely short trial duration, the absence of biopsy-level endpoints, and inconsistent control of exposure pattern and external load, should be read as the present limitations that motivate them.

## Conclusion

Caffeine is a reproducible acute ergogenic aid, but repeated peri exercise caffeine exposure has not been shown to consistently enhance or impair skeletal muscle adaptation in humans. The acute literature supports central adenosine receptor antagonism, potassium handling, and modest Ca²^+^ related contractile effects. The chronic literature remains mechanistically thin. Existing human studies are too short, too performance focused, and too sparse in endurance training and biopsy endpoints to resolve tissue level adaptation.

The available evidence is most consistent with partial mimicry and training quality mediation. Caffeine can reproduce selected acute signalling features in preclinical models and can improve session output in humans. It has not yet been shown to amplify human skeletal muscle remodelling when training load is matched. An attenuation model is plausible because animal and tissue studies suggest possible interference with protein synthesis, recovery, and selected adaptation endpoints. The model remains untested in humans.

**Figure 6:**
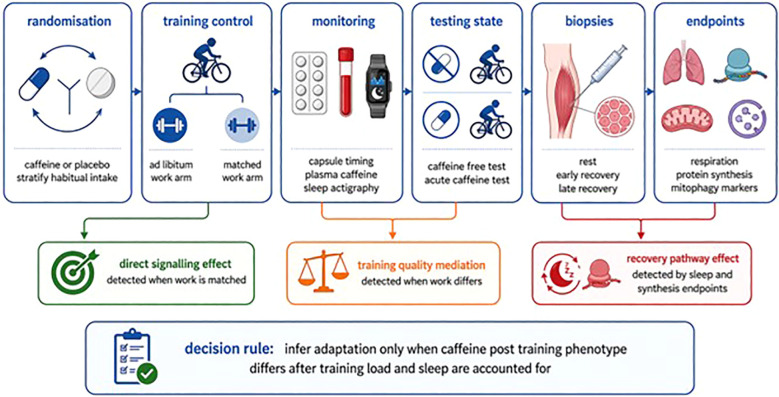
Trial design logic for separating direct signalling from training stimulus mediation. A direct signalling effect requires a tissue phenotype difference when work is matched. Training quality mediation requires a difference in accumulated work. A recovery pathway requires concurrent evidence from sleep or recovery phase endpoints.

Moderate caffeine intake before exercise can be justified for acute performance enhancement, but should not yet be presented as a strategy to enhance skeletal muscle adaptation. Resolving the chronic question requires human training trials that combine muscle biopsy endpoints, caffeine abstinent post training testing, objective sleep monitoring, and explicit control or modelling of external training load (**Figure 6**).

## Data Availability

The original contributions presented in the study are included in the article/supplementary material. Further inquiries can be directed to the corresponding author.
